# Prognostic Significance of Substance P/Neurokinin 1 Receptor and Its Association with Hormonal Receptors in Breast Carcinoma

**DOI:** 10.1155/2021/5577820

**Published:** 2021-10-13

**Authors:** Riffat Mehboob, Syed Amir Gilani, Amber Hassan, Sadaf, Imrana Tanvir, Shaista Javaid, Sidra Khalid, Sana Hasan, Humaira Waseem, Ahmad Alwazzan, Miguel Munoz

**Affiliations:** ^1^Research Unit, Faculty of Allied Health Sciences, University of Lahore, Lahore, Pakistan; ^2^Lahore Medical Research Center, LLP, Lahore, Pakistan; ^3^Lino Rossi Research Center, University of Milan, Milan, Italy; ^4^Center for Research in Molecular Medicine, The University of Lahore, Lahore, Pakistan; ^5^Department of Pathology, King Abdulaziz University, Rabigh, Saudi Arabia; ^6^Punjab Health Care Commission, Lahore, Pakistan; ^7^Fatima Jinnah Medical University, Lahore, Pakistan; ^8^Division of Gynecology Oncology, Faculty of Medicine, King Abdulaziz University, Jeddah, Saudi Arabia; ^9^Research Laboratory on Neuropeptides (IBIS), Virgen del Rocío University Hospital, Seville, Spain

## Abstract

Expression and immunolocalization of Substance P (SP)/Neurokinin-1 Receptor (NK-1R) in breast carcinoma (BC) patients and its association with routine proliferative markers (ER, PR, HER2/neu, and Ki-67) were evaluated. A cross-sectional study was performed on 34 cases of BC. There were 23 cases of group A (grade III), 8 of group B (grade II), and only 3 cases of group C (grade I). All samples were then processed for SP and NK-1R immunohistochemistry for few cases. 14/23 cases (61%) of group A, 7/8 cases (88%) of group B, and 2/3 (67%) cases of group C were SP positive. Overall, strong staining (≥10% tumor cells), labeled as “+3,” was observed in 9/14 (64.2%) cases of group A and 1/8 (12.5%) cases of group B. Moderate staining labelled as “+2” (in ≥10% tumor cells) was observed in 3/14 (21.4%) cases of group A and 4/8 (50%) cases of group B. Weak positive staining “+1” was observed in only 2/14 (14.28%) cases of group A, 2/8 (25%) cases of group B, and all 2/2 (100%) cases of group C. SP and NK-1R are overexpressed in breast carcinomas, and there is significant association between the grade of tumor and their overexpression.

## 1. Introduction

Breast cancer (BC) is the most common cancer in women all over the world with an incidence of approximately 2 million in 2018. The highest rate of BC was observed in Belgium with 113.2/100,000 women [1]. It can occur as a result of cells under the influence of estrogen multiplying and infringing on other tissues spreading to other regions of the body [2]. Worldwide, the occurrence of BC exceeds all female cancers with high mortality rates [3]. Despite the recent advances in BC therapy, the disease is still counted as a major health problem worldwide and remains an elusive disorder. In fact, poor prognosis, late diagnosis, and therapeutic challenges including the evolution of resistant cells and tumor heterogeneity have remained partly unavoidable and are considered as major challenges in the management of this disease.

A number of factors, such as histological grade, type and size of tumor, lymph node metastasis, estrogen receptor (ER), progesterone receptor (PR), and human epidermal growth factor receptor 2 (HER2/neu), influence the prognosis and response to the treatment of cancer [4]. Newer classification methods are also being developed that are based on immunohistochemical, genetic, and molecular findings. Availability of hormone (estrogen and progesterone) receptor markers marked the beginning of molecular classification about 30 years ago [5].

In BC, the malignant cells are enlarged with vacuolated cytoplasm and vesicular nuclei containing prominent nuclei. Most of the time, the stroma was found to be increased and degenerative in nature [6]. The grading of invasive BCs is an important factor in addition to the size and status of the lymph nodes [7]. Benign breast diseases especially fibroadenomas are also important, as some of them (30%) may lead to cancer [8]. The staging of BC is related to the size, location, and number of regional metastases to lymph nodes and sometimes is related to growth [9]. TNM stages IIB, IIIA, and IIIB are tumor stages that help in diagnosis [10].

There are various types of BC; they are classified as in situ and invasive. In situ carcinoma includes lobular carcinoma in situ (LCIS) and ductal carcinoma in situ (DCIS). Invasive carcinoma includes invasive lobular carcinoma (ILC) and invasive ductal carcinoma (IDC) [11]. According to the site, BC is divided into invasive and noninvasive lobular carcinoma. Invasive lobular carcinoma is the second most common type of BC, several histological subtypes exist, most of the tumors are classified as grade II, and the majority of grade III are among the nonclassified subtypes showing a disease-free region as compared to grade II [12]. The number of positive axillary lymph nodes and hormone receptor negative tumors increases among grade III tumors [13].

Further, a new study mandated the molecular classification of human BC by initially dividing BC into four major classes: luminal-like, basal-like, normal-like, and HER2 positive. Subsequently, luminal class was divided into luminal A and luminal B classes, thereby resulting in addition of a fifth class of BC [14]. According to the reported study, molecular subtypes of BC can be classified into luminal A (ER+/PR+/HER2-/low Ki-67), luminal B (ER+/PR+/HER2-/+/high Ki-67), HER2-overexpression (ER-/PR-/HER2+), and triple negative breast cancers/TNBCs (ER-/PR-/HER2-) [15].

Family history of both maternal and paternal relatives is important and has long been known as a risk factor for BC [16]. BC is commonly caused by low-penetrance genes that are involved in the DNA-repairing mechanism. DNA and chromosomal damage may also cause BC. The XRCC3Thr24 Met polymorphism is the most common gene associated to BC [17]. These are repair genes to rectify the DNA damage. These genes are involved in enhancing the cytotoxicity, apoptosis, p53 phosphorylation, and exposure to external factors that cause DNA damage [18]. BRCA1 and BRCA2 are abnormal genes that, when inherited, increase the estimated risk between 40 and 88% of BC. BRCA1 genes tend to develop BC at an early age [19]. In stage 2, about 54% of the women are diagnosed, while in stage 1, only 16% are diagnosed [20]. Sedentary life style, high dietary intake of fat, and obesity particularly in postmenopausal women may cause BC [21]. The use of alcohol is also another cause of BC [22].

Substance P (SP) is a small undecapeptide hormone [23] and the most abundant tachykinin (TK) peptide in the central nervous system of mammals [24]. Many physiological and pathological roles of this peptide have been noticed [25]. Munoz and Covenas [26] suggested a strong role of the SP-Neurokinin-1 receptor (NK-1R) system in the progression of carcinogenesis. SP mediates pain, neurogenic inflammation, and mitogenesis via interaction with its high-affinity receptor NK-1R, which is widely distributed throughout the body. NK-1R is widely distributed throughout the body. BC cells exhibit mRNA for the receptor of SP, NK-1, which is then involved in promoting the cell proliferation and, consequently, metastasis [27]. Additionally, SP is also involved in vasculogenesis, angiogenesis, and neoangiogenesis as observed in both *in vivo* and *in vitro* studies, an essential step towards invasion and metastasis [28, 29].

SP and NK-1R have been detected in tumor cells and in intra- and peritumoral blood vessels [26–28]; furthermore, SP has been shown to protect tumor cells from apoptosis [29]. The relevance of the SP/NK-1 receptor system has been specifically shown in pancreatic cancer, where SP is involved in pancreatic cancer proliferation, neoangiogenesis, and migration of pancreatic cancer cells, and SP receptor antagonism has been shown to reverse these alterations [26, 29–31]. These findings suggest that elevated SP can be detrimental in cancer and suggest that NK-1R antagonism can be beneficial in cancer treatment.

To our knowledge, it is the first study to report the expression and distribution of SP in BC and to suggest a strong association of its expression with the progression of disease and its association to routine proliferative and hormonal markers. Thus, the aim of this study is to evaluate the expression of SP/NK-1R and its relationship with tumor type and clinicopathological parameters of BC patients. Furthermore, the relationship between the SP/NK-1R and proliferative markers was investigated.

## 2. Material and Methods

We have followed the same methods for data collection and immunohistochemistry as done in our previous study [30]. The study setting was the Faculty of Allied Health Sciences, the University of Lahore, Lahore, Pakistan. A total of 34 formalin-fixed paraffin-embedded (FFPE) blocks of BC were included. Medical and personal history of patients consisted of age, span of disease, tumor site/size, progression of disease, staging/grading, etc. Age range was 20-80 years. For collection of data, we followed the American Joint Committee for Cancer Staging and End Results Reporting. All the parameters of the Declaration of Helsinki were respected in this study. Classification of the tumor was based on WHO criteria such as well differentiated (WD), moderately differentiated (MD), and poorly differentiated (PD) breast carcinoma for grade I, grade II, and grade III, respectively. All the slides were routinely stained with hematoxylin-eosin to assess the morphology of cells and proper classification of cases. These were interpreted by two histopathologists. Data were entered in SPSS 24.0. A chi square test was applied to check the association between the SP and NK-1R expression (positive and negative stains) and other parameters.

### 2.1. ER, PR, HER2, and Ki-67staining

Immunohistochemistry (IHC) for ER, PR, HER2/neu, and Ki-67 was accomplished on FFPE tissue segments as part of the routine clinical assessment of these cases. Antibodies against ER, PR, HER2, and Ki-67 were obtained from Dako, Denmark, and used in concentrations as per the manufacturer's protocol. Lobular and ductal normal areas of the breast were used as the control for ER, PR, and HER2 IHC, whereas the appendix tissue was set as the control for Ki-67. Olympus (Model U-DO3) was used for microscopy.

### 2.2. Substance P/NK-1R Immunohistochemistry (IHC)

FFPE sections of 4 *μ*m were deparrafinized with xylene and decreasing grades of alcohol and washed in distilled water and then Phosphate Buffer Saline (PBS). These sections were pretreated with a citrate buffer in microwave and were allowed to cool for at least 20 minutes. Washings in distilled water and PBS were done before 3% H_2_O_2_ (30 minutes) to block the endogenous peroxidase activity. SP antibody (BioGenex) in dilution 1 : 100 and NK-1R antibody (Abcam) in 1 : 100 dilution were applied to the sections for 45-50 minutes in a humid chamber. The washing step in PBS was done for 10-15 min. Slides were then incubated with secondary antibody Horse Radish Peroxidase (HRP) (Abcam ab6789) for 45-50 minutes and washed again with PBS (1x, pH 7.4) (10-15 minutes). 3,3′-Diaminobenzidine (DAB) DAB plus, K3468, Dako, Denmark, was applied for 5-10 minutes and counter stained with Mayer's hematoxylin for 2 minutes. FFPE sections were dipped in increasing grades of alcohol and then xylene for 5 minutes each. DPX mounting medium was used, and slides were cover slipped. Methods are similar to one of our previous studies on oral squamous cell carcinoma [30].

### 2.3. Grading of IHC

Cell counting at 10x and 40x was done for the evaluation of protein expression, and counts were made as in our previous study (Table 1) [31]. Scoring for ER, PR, HER2, and Ki-67 was done by the Allred method proposed by Qureshi and Pervez [32] (Table 2). No protein overexpression or membrane staining in <10% tumor cells was labeled as score “0” and considered negative for SP/NK-1R protein overexpression. Faint/weak staining (in ≥10% of tumor cells) was given the “+1” score, moderate staining as “+2,” and strong staining as “+3.”

## 3. Results

### 3.1. SP/NK-1R Expression

**Figure 1:**
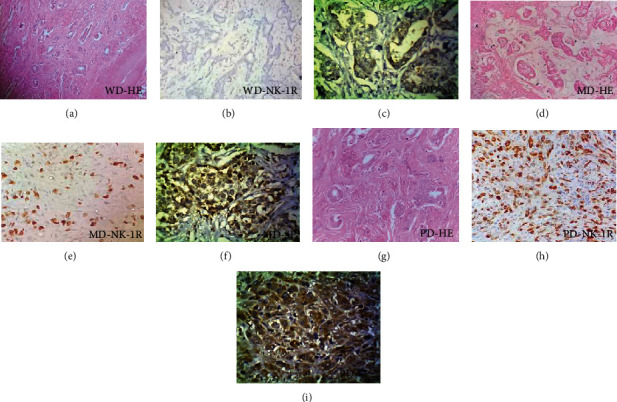
BC at 40x: (a) WD-BC hematoxylin-eosin staining (b) grade 1, NK-1R negative (c) SP weakly positive +1; (d) MD-BC hematoxylin-eosin staining (e) MD, grade 2, NK-1R moderately positive, +2, 40% cells showing positive stain (f) MD, grade 2, SP moderately positive, +2; (g) PD-BC hematoxylin-eosin staining (h) PD, grade 3, strongly SP positive, +3, 90% SP positive cell (i) PD, grade 3, strong positive, +3, 85% cells showing positive stain.

**Figure 2:**
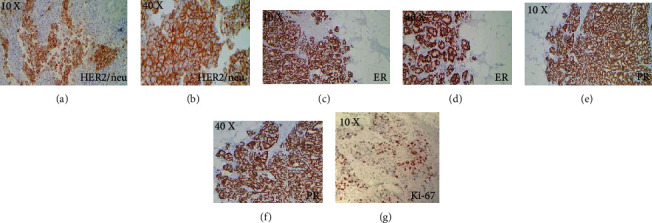
Staining with routine diagnostic markers for BC: (a, b) HER2 strongly positive, complete membranous staining in more than 80% of tumor cells, 10x and 40x; (c, d) ER strongly positive, nuclear staining in 95% of tumor cells, ALLRED score 5 + 3, 10x and 40x; (e, f) PR strongly positive, nuclear staining in 95% of tumor cells, ALLRED score 5 + 3, 10x and 40x; (g) Ki-67 proliferative marker, strongly positive in 30% of tumor cells, 10x and 40x.

Expression of SP and NK-1R was detected to be cytoplasmic. Expression of SP showed 68% (23) of the BC cases to be positive (Table 2). Cases of well-differentiated (WD) carcinoma had clear cells with cytoplasm and nucleus (Figure 1(a)), and most of them (66.6%) were SP/NK1R positive (Figure 1(c), Table 2). In moderately differentiated (MD) cases, little morphology of cells has been disrupted, but so far, they can be recognized (Figure 1(d)). In poorly differentiated (PD) cases (Figure 1(g), 14 cases, 60.8%), maximum intensity (+3) of SP was observed (Figure 1(i); Table 2), whereas (7 cases, 87.5%) (Figure 1(f), Table 2) MD with +2 intensity of SP expression and low intensity (+1) was seen in WD cases (2 cases) (Figure 1(c), Table 2). In poorly differentiated cases, the cell morphology was extremely distorted, and cells could not be simply distinguished (Figure 2). Immunohistochemical staining for NK-1R was completed in a small number (6) of core biopsies. The expression of NK-1R was similarly found to be related with the progression of BC. Its expression was high in MD and PD cases (Figures 1(e) and 1(h)) while almost negative in WD cases (Figure 1(b)).

### 3.2. Association of SP and Patient Characteristics with Clinicopathological Features of BC Patients

The maximum number of the SP-positive cases 19/23 (82.6%) belonged to the age group of <60 years. 12/23 (52.17%) SP-positive cases belonged to premenopausal females and 11/23(47.82%) to postmenopausal females. In most cases, 15/23 (65.2%) had tumor sizes ranged between 2 and 5 cm. 14/23 (60.8%) cases of PD or grade III (group A), 7/23 (30.43%) cases of MD or grade II (group B) and 2/23 (8.69%) cases of WD-BC or grade I (group C) were SP positive. According to the TNM staging, 15/23 (65.2%) SP-positive cases had PT2 stage. According to tumor type, 15/23 (65.2%) SP-positive cases were invasive ductal carcinoma. Distant metastasis was absent in the majority (18/23, 78.26%) of the SP-positive cases. Axillary lymph node metastasis was also absent in 15/23 (65.2%) cases (Table 2).

#### 3.2.1. Distribution of Positive Cases of SP according to the BC Classification

Interpreting from the division of BC, 5/23 (21.73%) SP-positive cases belonged to the luminal A group (ER/PR+, HER2-), 14/23 (60.8%) cases belonged to the luminal B (ER/PR+, HER2+) group, and 4/23 (17.39%) cases belonged to the ER/PR- and HER2+ group of BC (Table 3).

#### 3.2.2. SP Association with ER, PR, HER2, and Ki-67

ER was SP positive in 19/23 (82.6%) cases; PR was positive in 17/23 (73.9%) and HER2 was positive in 18/23 (78.2%) SP-positive cases (Figures 2(a)–2(f)). Ki-67 was positive in all the cases (Figure 2(g)) (Tables 2, 4, and 5). *H* scoring; Allred scoring; expressions of SP, ER, PR, and HER2/neu; and intensities of all stains are mentioned in Tables 4 and 5.

## 4. Discussion

For the first time, it is demonstrated that SP is not only overexpressed but also involved in the progression of BC. It is found to be associated with poor prognosis and advancement of disease as reported by a previous study [27]. BC cells may release SP after binding to its receptor, NK-1R, as a possible mechanism; it may lead to proliferation [27], migration [29], and angiogenesis [33]. SP may also cause inflammation by enhancing the permeability of the blood-brain barrier (BBB) [34]. Subsequently, BC cells migrate and metastasize.

Similar findings were observed in our study except that we evaluated SP and NK-1R both in the tissue, but in a previous study [35], only NK-1R was evaluated in the tissue. There is little contradiction in SP evaluation: in our study, we observed an increased expression with an increasing grade of the tumor, while in a previous study, no difference among the grades was observed and it was only performed on the serum. We revealed the SP expression in all grades of BC which was commonly positive, and the intensity increased with advancing grade. It demonstrates that SP expression is associated with the poor prognosis and aggression of this illness. Our outcomes are in concordance with the earlier studies on BC, which showed SP overexpression [27]. SP discharge from BC cells in response to nociceptive stimuli, whose consequences result in proliferation [27], metastasis, and vasculogenesis [29] by functioning of the autocrine role and causes inflammation by the paracrine role. SP raises the absorptivity of the blood-brain barrier (BBB) [33, 34]. An advanced grade of BC showed higher intensity of SP expression; they can be involved in metastasis.

When more SP is released, it can decrease the apoptosis subsequently [36] by modulating the immune markers IL4, IL6, and IL10 [37], resulting in unrestrained cell division, cell progression, and prominent cancer metastasis. All these mechanisms are carried by increased cellularity in human tenocytes [38] resulting in binding of SP to NK-1R. SP has been described to phosphorylate the AKT (antiapoptotic protein kinase) [39]. SP has been studied in bone marrow stem cells showing proliferative effects [40], but it has to be explored extensively in cancer.

Previously, we had demonstrated the immunohistochemical expression of SP in the sudden fetal and infant deaths and neuropathology [41–44]. We also established SP expression in oral squamous cell carcinoma (OSCC), where a strong expression of SP was found to be related with the progression of OSCC and aided as a diagnostic marker [30]. It was directly related to the grade of cancer, i.e., intensity of expression increased with the increasing grade. An *in silico* analysis by us also revealed the possible involvement of the Tachykinin 1 (Tac1) gene, a gene for SP, in cancer [45]. In another study, the SP/NK-1R system is found to be associated with colorectal cancer progression and prognosis [46].

The Tachykinin family is the largest peptide family; its members bind to G-protein coupled receptors at the cells of destination. Hence, a signaling cascade is initiated, leading to mitogen-activated protein kinase activation, mobilization of calcium, and phosphoinositide hydrolysis. The tumor microenvironment plays a crucial role in this regard, and SP carries its role by binding to NK-1R [33]. SP is found to be important for the viability of cancer cells, and NK-1R has been observed to be more expressed in these cells [47]. SP and NK-1R expression has been found to be associated with the progression of several diseases [26, 48]. Our study is in accordance with these studies, and we observed an overexpression of SP in grade III and an intermediate expression in grade II.

Overexpression of SP and NK-1R was also observed in the precancerous epithelium, and it was proposed that it has contribution towards early carcinogenesis by increasing cell growth and cell division [49]; however, in the current study, this trend was found in a later stage of disease. NK-1R antagonists may inhibit cellular growth, proliferation, and metastasis. It may have a therapeutic role for cancer treatment by inhibiting neoangiogenesis and vascularization. It may be explored for potential as antitumor drugs [26]. It may block the signal transduction network in the cancer microenvironment and reduce the proliferation of tumor cells [48]. By contrast, NK-1R antagonists act in a concentration-dependent manner and counteract the pathophysiological functions of SP. So, NK-1R antagonists may inhibit BC cellular growth, proliferation [27], and migration (for invasion and metastasis) [29]. It may have a therapeutic role for cancer treatment by inhibiting neoangiogenesis and vascularization. It may be explored for potential as antitumor drugs [26]. It may block the signal transduction network in the cancer microenvironment and reduce the proliferation of tumor cells [48].

BC cells not showing HER2/neu amplification and not expressing estrogen/progesterone receptors are named triple-negative BC (TNBC) cells. TNBC represents 10-15% of all BC and is associated with an aggressive clinical course. TNBC patient prognosis, survival, and response to current therapies are poor, and for this reason, it is crucial to search for new therapeutic targets in TNBC to develop new therapeutic strategies. One of these targets is the Neurokinin-1 receptor (NK-1R). It is well known that the SP/NK-1R system is involved in cancer progression. TNBC cells overexpress the NK-1R, and after binding to this receptor, SP promotes the proliferation/migration of TNBC cells. Nonpeptide NK-1R antagonists (e.g., aprepitant) are known to exert, via the NK-1R, an antitumor action; TNBC cells die by apoptosis. The review report conducted by Miguel Muñoz updates the data on a promising therapeutic innovation of NK-1R antagonists for the treatment of TNBC patients [50]. The patient remained in good health, with no side effects, and the tumor volume also decreased [51]. Further research and clinical trials must be carried out in order to fully reveal the beneficial effects of NK-1R antagonists in the treatment of patients suffering from BC. NK-1R antagonists can help in inhibition of various cancers by blocking angiogenesis [52]. Recently, we have proposed the NK-1R antagonist, aprepitant, as a therapeutic strategy for inflammation and respiratory symptoms in COVID-19 infection [53–55]. It has also been reported in our recent findings in dental inflammation and pain [56] as well as being associated with miscarriages [57]. We emphasize further research on the SP/NK-1R pathway in breast cancer as well as other cancers.

## 5. Conclusion

We hereby conclude that increased intensity and overexpression of Substance P and NK-1R is associated with poor prognosis in BC. SP/NK-1R may also be explored further as a potential diagnostic biomarker for BC to differentiate the grades.

## Figures and Tables

**(a):** 

Allred	Cell stain		Proportion score
	%	Score (3)	
Negative	0	0	0
Weak positive	1	1	1
Moderate positive	1-10	2	2
Strong positive	10-33	3	3
	33-66		4
	66-100		5

**(b):** 

Sum of proportion score and intensity score	
Negative	0-2
Positive	3-8

**Table 2:** Clinicopathological features of studied patients.

*n* = 34	SP+ (23)	SP- (11)	Total (34)	*P* value
Age(years)				
>60	4 (17.39%)	1 (9.09%)	5 (14.7%)	NS
<60	19 (82.6%)	10 (90.9%)	29 (85.29%)	
Menopause status				
Pre	12 (52.17%)	7 (63.63%)	19 (55.88%)	NS
Post	11 (47.82%)	4 (36.36%)	15 (44.11%)	
Tumor size(cm)				
<2	3 (13.04%)	0	3 (8.82%)	NS
2-5	15 (65.2%)	8 (72.7%)	23 (67.64%)	
>5	5 (21.73%)	3 (27.2%)	8 (23.52%)	
Grade				
I (well diff)	2 (8.69%)	1 (9.09%)	3 (8.82%)	NS
II (mod)	7 (30.43%)	1 (9.09%)	8 (23.52%)	
III (poor)	14 (60.8%)	9 (81.8%)	23 (67.64%)	
TNM				
PT1	4 (17.39%)	1 (9.09%)	5 (14.7%)	NS
PT2	15 (65.2%)	8 (72.7%)	23 (67.64%)	
PT3	2 (8.69%)	2 (18.2%)	4 (11.76%)	
PT4	2 (8.69%)	0	2 (5.88%)	
Tumor type				
IDC	15 (65.2%)	5 (45.5%)	20 (58.82%)	0.005^∗^
DCIS	1 (4.34%)	6 (54.5%)	7 (20.58%)	
ILC	2 (8.69%)	0	2 (5.88%)	
IDC+DCIS	5 (21.7%)	0	5 (14.7%)	
ER status				
+ve	19 (82.6%)	9 (81.81%)	28 (82.35%)	NS
-ve	4 (17.39%)	2 (18.18%)	6 (17.64%)	
PR status				
+ve	19 (82.6%)	9 (81.81%)	28 (82.35%)	NS
-ve	4 (17.39%)	2 (18.18%)	6 (17.64%)	
HER2/neu status				0.017^∗^
+ve	18 (78.26%)	4 (36.4%)	22 (64.7%)	
-ve	5 (21.7%)	7 (63.4%)	12 (35.29%)	
Ki-67 status				
+ve	23 (100%)	7 (63.4%)	30 (88.23%)	0.002^∗^
-ve	0	4 (36.4%)	4 (11.76%)	
Distant metastasis				
Present	3 (13.04%)	1 (9.09%)	4 (11.76%)	NS
Absent	18 (78.26%)	9 (81.8%)	27 (79.41%)	
Unknown	2 (8.69%)	1 (9.09%)	3 (8.82%)	
Lymph node metastasis (axillary)				
1-3 lymph nodes	2 (8.69%)	2 (18.2%)	4 (11.76%)	NS
>4 lymph nodes	6 (26.08%)	3 (27.3%)	9 (26.47%)	
Absent	15 (65.2%)	6 (54.5%)	21 (61.76%)	

^∗^NS: nonsignificant.

**Table 3:** Clinical classification of breast cancer cases and its association with SP expression.

Types of breast cancer	SP+	SP-
Luminal A (ER/PR+, HER2-)	5	7
Luminal B(ER/PR+, HER2+)	14	2
ER/PR-, HER2+	4	2
Total cases	23	11

**Table 4:** Expression and scoring of ER, PR, and HER2 in SP-negative breast cancer cases.

SP-negative cases (*n* = 11)													
Age (years)	Grade	Histoopinion	Expression	% of cell stain	Intensity of stain	Allred score	Expression	% of cell stain	Intensity of stain	Allred score	Expression	TNM	Size
			ER status				PR status				HER2 status		
50	1	DCIS-CB	+++	5	3	8	++	4	3	7	−	PT1	>5
40	3	IDCIS	−	−	−	−	−	−	−	−	+	PT3	>5
40	3	IDCIS	+	−	−	−	+	−	−	−	−	PT2	2-5
37	3	IDCIS	+	2	2	4	++	3	3	6	+++	PT2	2-5
42	3	IDC	+++	5	3	8	+	2	2	4	+++	PT2	2-5
47	3	IDC	++	3	3	6	++	4	3	7	−	PT2	2-5
35	2	DCIS (nipple involved)	−	−	−	−	−	−	−	−	+	PT2	2-5
40	3	IDCIS	+	−	−	−	+	−	−	−	−	PT2	2-5
57	3	IDC	++	2	2	4	+	6	−	−	−	PT2	2-5
72	3	IDC	++	4	2	6	+	−	−	−	−	PT3	>5
57	3	IDC	+++	5	3	8	++	4	3	7	−	PT2	2-5

**Table 5:** Expression and scoring of ER, PR, and HER2 in SP-positive breast cancer cases.

SP-positive cases (*n* = 23)															
Age (years)	Grade	Histoopinion	SP expression	*H* score	Expression	% of cell stain	Intensity of stain	Allred score	Expression	% of cell stain	Intensity of stain	Allred score	Expression	TNM	Size (cm)
					ER status				PR status				HER2 status		
33	2	IDC	+++	140	−	−	−	−	−	−	−	−	++	PT2	<2
58	3	IDC=DCIS	+++	190	+++	5	3	8	+++	5	3	8	+++	PT4	2-5
57	3	IDC	+++	240	+++	5	3	8	+++	5	3	8	+++	PT2	2-5
34	3	IDC	+++	240	+	2	2	4	++	4	2	6	−	PT2	<2
50	3	IDC+DCIS	+++	150	+++	5	3	8	++	4	2	6	++	PT4b	2-5
62	3	IDC+DCIS	++	120	+	2	3	5	++	−	−	6	++	PT2	2-5
54	3	IDC	+++	210	+++	5	3	8	+++	5	3	8	+++	PT2	2-5
33	2	IDC	++	160	+++	5	3	8	++	4	3	7	++	PT2	2-5
37	3	IDC+DCIS	+++	210	++	4	2	6	+++	5	3	8	+++	PT1c	>5
67	2	IDC+E-DCIS	+	140	+	3	1	4	−	+	−	−	−	PT3	2-5
63	1	DCIS	+	60	+	2	2	4	++	3	3	6	−	PT1	>5
32	3	IDC	+	180	+++	5	3	8	+++	5	3	8	+++	PT2	<2
33	3	IDC	+	70	+	3	2	5	++	3	3	6	−	PT2	2-5
42	2	ILC	+	150	+++	5	3	8	++	4	3	7	++	PT2	>5
57	3	IDC	++	140	−	−	−	−	−	−	−	−	++	PT2	2-5
37	3	IDC	++	150	−	−	−	−	−	−	−	−	+	PT2	2-5
47	2	IDC	++	180	+	3	1	4	−	+	−	3	+	PT2	2-5
34	3	IDC	+++	160	+	2	2	4	+	3	1	4	−	PT1	>5
50	3	IDC	+++	210	+	2	2	4	++	5	2	7	++	PT3	2-5
67	1	IDC	+	160	+++	5	3	8	++	4	3	7	++	PT2	2-5
42	3	IDC	+++	240	+++	5	3	8	++	4	3	7	++	PT1c	>5
32	2	ILC	++	180	+++	5	3	8	+++	5	3	8	++	PT2	2-5
50	2	IDC	++	160	−	−	−	−	−	−	−	−	+	PT2	2-5

## Data Availability

The data will be furnished upon request.

## Additional Points

*Key Points*. Immunohistochemical expression of Substance P and Neurokinin-1 receptor in breast carcinoma tissue was evaluated. It was strongly expressed in grade III, with maximum intensity. It may be investigated further for its role as a prognostic and a diagnostic marker. The therapeutic potential of Neurokinin-1 receptor antagonists must be explored.
